# Measurement of anti-allergic effects using flow-through piezoelectric biosensors based on the mast cells exocytosis

**DOI:** 10.1007/s00604-026-08084-6

**Published:** 2026-04-23

**Authors:** Zdenka Fohlerova, Laila A. Daliberto, Maria Perpetua F.M. Del Lama, Jan Pribyl, Zuzana Koselova, Petr Skladal, Zeki Naal, Rose M.Z.G. Naal

**Affiliations:** 1 Department of BioMolecular Sciences, Faculty of Pharmaceutical Sciences, University of Sao Paulo, Ribeirao Preto, Brazil; 2 Department of Biochemistry, Faculty of Science, Masaryk University, Kamenice 5, Brno, 625 00 Czech Republic; 3 Nanobiotechnology Core Facility Masaryk University, CEITEC, Brno, Czech Republic; 4 Department of Microelectronics, Brno University of Technology, Brno, Czech Republic; 5 Department of Biomedical Engineering, Brno University of Technology, Brno, Czech Republic; 6 National Institute of Science and Technology in Bioanalytical, UNICAMP, Campinas, Brazil; 7 Department of Microelectronics, FEEC, Brno University of Technology, Technicka 10, Brno, 616 00 Czech Republic

**Keywords:** Cell-based biosensor, Degranulation, Quartz crystal microbalance, Atomic force microscopy

## Abstract

IgE-mediated degranulation of mast cells is involved in many allergic diseases due to the proallergic and proinflammatory substances released from granules when they become activated. The initial step of sensitization requires IgE antibodies to bind to high affinity FcεRI receptors. The following activation of cells becomes triggered by multivalent antigen-induced oligomerization of the IgE/FcεRI complexes. The activation reaction is accompanied by microstructural changes of the cells, which can serve as signal-generating event. To monitor such events in real time, a piezoelectric (PZ) quartz crystal resonator sensor was adopted to provide semi-quantitative and qualitative information relating to the structural changes and dynamics of the process. The sensitized RBL-2H3 mast cells were cultured on poly-L-lysine coated gold surface of quartz crystal integrated in the flow cell and connected with the detector providing resonance frequency and resistance of the piezosensor. The activation of mast cells by antigen–dinitrophenyl conjugated to bovine serum albumin, was continuously monitored for several hours. Furthermore, quercetin, as the efficient inhibitor of the degranulation process, was tested in micromolar concentrations. The observed PZ response was confirmed using atomic force microscopy for imaging topography and morphological changes of the mast cells fixed at different stages of the degranulation. The results from the constructed PZ biosensor proved to be sensitive, fast, and label-free tool for screening of anti-allergic drugs.

**Graphical Abstract:**

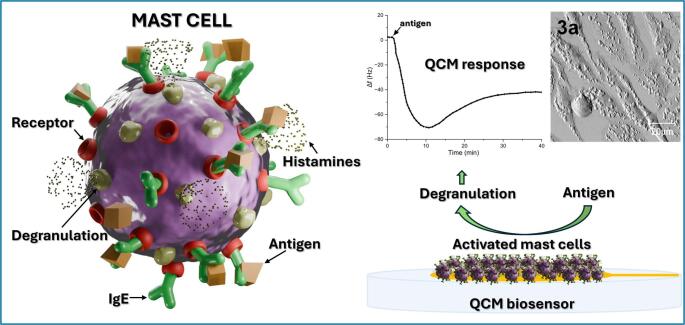

## Introduction

Exocytosis is a complex cellular event involving fusion of secretory vesicles with the cytoplasmatic membrane. Subsequently, the cell can release newly synthesized membrane impermeable substances to the extracellular space and biomolecules as proteins and lipids can be inserted into the plasma membrane of surrounding cells. The exocytosis of many secretory cells, such as mast, chromaffin, and neuronal cells, is strictly regulated and it differs regarding the type and size of secretory vesicles, content, kinetics, and the process of release. There is no doubt that the activation of the relevant signaling pathway triggers the final membrane fusion, followed by the release of vesicular content and the accompanying, more or less extensive, cytoskeletal rearrangement [1, 2]. Mast cells (MC) have been recognized for their crucial role in the development of allergic and inflammatory diseases [3, 4]. When activated, the release of compounds such as histamine, heparin, proteases, tumor necrosis factor, cytokines and prostaglandins occurs. Here, the RBL-2H3 cells (mucosal mast cells) as well-defined and commonly studied cell line for the robust regulated exocytosis were chosen for experiments.

**Fig. 1:**
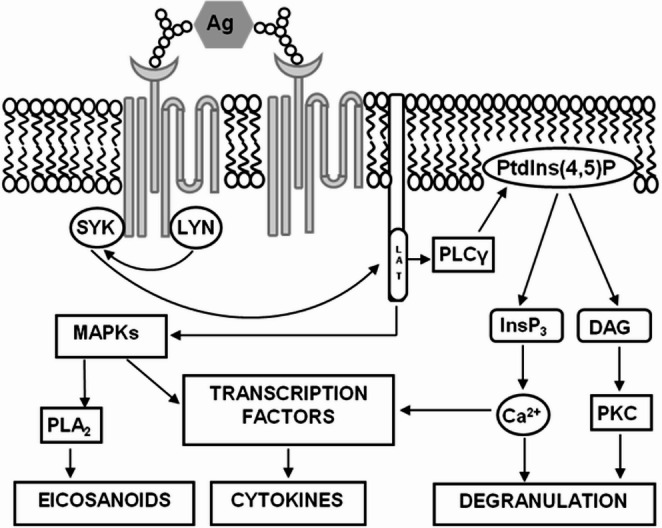
Signaling cascade in mast cells activated by antigen via the IgE-FcεRI receptors in the cellular membrane. The cross-linking of IgE-receptor complexes by antigen initiates the activation of signaling pathway, in which the phospholipase Cγ (PLCγ) cleaves phosphatidylinositol 4,5-diphosphate (PtdIns(4,5)P_2_) to diacylglycerol (DAG) and inositol triphosphate (InsP_3_). The crucial role is the InsP_3_-induced calcium release from the storage and activation of protein kinase C (PKC), both subsequently promoting degranulation. Later activation of transcription factors and phospholipase A_2_ (PLA_2_) lead to the production of cytokines and prostaglandins. (adapted from [30])

Allergic response of the adopted cell line is primarily mediated by the IgE dependent pathway (IgE-bound antigen) but some complement components, pathogens and physical stimuli are able to induce the degranulation in the IgE independent way [5, 6]. In the IgE dependent way (Fig. 1), the cells become sensitized by the IgE antibodies that bind the high affinity FcεRI receptors present on the outer cellular membrane. The cross-linking of at least two receptor/IgE complexes by the oligovalent antigen initiates a cascade of biochemical events resulting in the cellular degranulation (“early phase” of the allergic reaction), followed by “late phase” lasting hours and involving the recruitment and activation of inflammatory cells [7]. Apart from degranulation, the activation of mast cells is accompanied by alkalinization of the secretory granules, increased level of intracellular calcium, and the generation of reactive oxygen species (ROS) in the cytoplasm [8, 9]. Exocytosis is further associated with membrane ruffling and cytoskeletal rearrangement leading to the increased cellular adhesion and spreading [10, 11]. The degranulation of mast cells, in its complexity, might provide countless targets for pharmaceutical substances to avoid the allergic response [12, 13]. The described microstructural changes seem very promising for monitoring with piezoelectric sensors, eventually providing a novel approach to cell-based biosensing.

Furthermore, substances exhibiting the desired effect at any level of activation can be e.g. antagonists of receptors or inhibitors of enzymes. In our work, the polyphenolic flavonoid quercetin was adopted to block the degranulation. After binding of quercetin, the cell membrane becomes stabilized due to the blockade of calcium effect and thus the release of pro-allergic mediators cannot occur [14]. This effect comes from the inhibited expression of inflammatory cytokines through attenuation of NF-κB and p38 MAPK regulatory pathways [15]. Consequently, the release of histamine is blocked, which is typical for other flavonoids [16]. Therefore, searching for the new, effective, and clinically available inhibitors, particularly natural ones, appears very promising. Recently, 28 natural product extracts inhibited human IgE-FceRI binding and exhibited anti-histamine effects, as tested using protein biochips [17]. 

The quantification of cellular degranulation usually relies on fluorescence methods based on the specific fluorescence probes for intracellular calcium ions and reactive oxygen species (ROS) or measurement of fluorescence product of an involved enzyme, β-hexosaminidase [8, 18]. Such end-point methods are time-consuming, utilize labels, require the lysis of cells, and each step must be carefully optimized. The introduction of real-time and label-free methods, such as piezoelectric (PZ) quartz crystal resonator sensors, provides a useful tool for the semiquantitative evaluation of mass and viscoelastic changes combined with membrane-related phenomena. In this way, the screening of pharmaceutical substances appears very feasible [19–21]. However, for simple bioanalytical detection of allergens, electrochemical impedance techniques utilized paper-based sensors for peanuts protein [22] and milk casein [23]. The screen-printed sensor with mast cells was applied for bacterial spoilage quorum signaling molecules (N-acyl-homoserine-lactones) [24]. On the other hand, the screening of anti-alergic drugs was carried out using multiparallel surface plasmon resonance imaging custom system [25]. 

PZ sensors utilize a thin quartz crystal plate coated with metal electrodes on both sides. If a suitable sinusoidal potential becomes applied on the electrodes, the quartz plate starts to vibrate at its resonance frequency. The frequency shifts either when the vibration is damped due to viscoelastic changes or after mass loading at the sensing surface, and these effects can be followed continuously in real time. The contribution of the mass and viscoelasticity of the adjacent biomolecular or cellular layer to the resonance frequency change can be distinguished by measuring additional parameters such as the resistance or dissipation factor of the PZ resonator [26]. Measuring of both frequency and dissipation was utilized for monitoring of high K^+^- induced exocytosis in the NG 108 − 15 and PC12 cell lines [27]. The vesicular release and retrieval were detected as a small steep increase of the frequency followed by immediate decreasing within 30 s. The dissipation parameter behaved inversely. However, this type of exocytosis is not obviously linked with an extensive cytoskeletal change as evident at the antigen-stimulated degranulation of mast cells monitored using microelectrode array [28]. The antigen-induced degranulation was recorded as the transient increase of resistance within 30 min, followed by a slow return to the baseline within 4 h.

The goal of our work is detection of the dynamics of the antigen-induced exocytosis of the IgE-sensitized RBL-2H3 cells in real-time, sensitively and without any labels using the flow-through PZ quartz crystal microbalance system. The degranulation course differs from others because instead of pure mass changes due to exocytosis/endocytosis, the cell morphology and mechanical properties of membrane are measured. Quercetin, the potential inhibitor of cell degranulation, was tested as a blocker of the degranulation. For imaging of the sensing surfaces, fluorescence and classic visual microscopies provide 2D images of the sample, an advanced FRET-based approach was reported for mast cell degranulation [29]. However, atomic force microscopies (AFM) provide higher resolution and 3D topographic views of the sample surface. In reality, fluorescence / visual microscopies are performed in advance in order to better or more precisely select the sample regions promising for the following AFM imaging. Here, AFM served for imaging of morphological and membrane changes of activated RBL-2H3 mast cells, thus confirming results from the PZ sensor.

The proposed PZ biosensor has the great potential for screening of anti-allergic drugs and studying the dynamics of exocytosis.

## Experimental setup

### Reagents

Mouse monoclonal anti-2,4-dinitrophenyl (DNP) IgE was purified from ascites cells as previously described [31]. Poly-L-lysine (PLL) was purchased from Sigma. MEM cultivation medium was from Atlanta Biologicals, USA. BSA conjugated with an average of 15 DNP groups (DNP-BSA) was prepared as previously described [32], dissolved in PBS and diluted to the required final concentration in the MEM medium. Quercetin was purchased from Sigma and dissolved in DMSO.

The 5 MHz quartz crystals (Stanford Research System, USA) were washed with acetone and coated with 0.1 mg mL^− 1^ solution of PLL for 3 h. The used crystals were regenerated (max. thrice) using the piranha solution (H_2_SO_4_:H_2_O_2_ in a ratio of 3:1).

### Cell culture

The RBL-2H3 (rat basophilic leukemia) cells were maintained in the MEM medium supplemented with 20% fetal bovine serum and 10 µg mL^− 1^ gentamicin. The cells were harvested with trypsin/EDTA solution (0.25%), sensitized by the addition of 5-fold molar excess of anti-DNP-IgE in the culture medium, and seeded on the piezocrystal overnight to obtain a confluent coating.

### β-Hexosaminidase release assay

The β-hexosaminidase (β-hex) release assay was performed as previously described [18]. The IgE-sensitized cells (1.5 × 10^5^ cells mL^− 1^) attached to microplate wells were treated with 100 µL of each concentration of quercetin prepared in Tyrode’s buffer (1.0 mg mL^− 1^ stock solution in DMSO), followed by the addition of 100 µL of the antigen (DNP-BSA; 0.1 µg mL^− 1^) at 37 °C, for 60 min. Finally, 100 µL of *β*-hexosaminidase substrate, 4-methylumbelliferyl-N-acetyl-β-D-glucosaminide (1.2 mmol L^− 1^), prepared in Tyrode’s buffer, was added and the cells were incubated for 30 min at 37 °C. The reaction was stopped by placing the cells on ice and the β-hexosaminidase activity was accessed by the measurement of its fluorescent product, umbelliferon, in a microplate reader (BioTek, Synergy HT, Winooski, Vermont, USA) using 360 nm excitation and 450 nm emission filters. The percentage of released β-hex was calculated using Eq. 1 and it was based on the total amount of released β-hex from cells disrupted with the surfactant Triton X-100. The spontaneous release was determined from cells in Tyrode’s buffer in the absence of antigen stimulation. IC_50_ values were determined graphically.

1 $${\%}\beta\;hex=\frac{S-N}{T-N}{\times\;100}$$

The symbols include *S* sample, fluorescence regarding released β-hex from (+) or (-) DNP-BSA treated cells; *N* normal, fluorescence from vehicle (Tyrode’s buffer); *T* total, fluorescence regarding released β-hex from Triton X-100 disrupted cells. The concentration of the tested compound that caused 50% of β-hex inhibition (IC_50_) was obtained using the Origin 8.0 software (Microcal, USA).

### Measurements of exocytosis using piezosensors

The QCM200 system (Stanford Research System, USA) comprises crystal holder, a controller, crystal oscillator electronics, and the flow cell adapter attach to the crystal holder to create a small volume stagnation point flow cell. The medium was maintained at 37 °C and presaturated with CO_2_ (5%). Flow rate was set to 70 µL min^− 1^. The crystal holder with IgE-sensitized mast cells was connected to the equipment and the flow system. As soon as the frequency and resistance responses were stabilized under the continual flow of the thermostated (37 °C) and CO_2_ (5%) presaturated medium, antigen was injected through the FIA system into the chamber using the concentration range from 10^− 7^ to 1 µg mL^− 1^. The signal was recorded during the following hours. In an inhibition assay, the medium was supplemented with various concentrations of quercetin (0.1–150 µmol L^− 1^) and the antigen (0.1 µg mL^− 1^) was injected afterwards as described above. Data was fitted with logistic function.

### AFM imaging

The RBL – 2H3 cells were sensitized by IgE and stimulated by 0.1 µg mL^− 1^ DNP-BSA, and after the desired incubation period fixed with the mixture of 4% formaldehyde and 0.1% glutaraldehyde for 20 min. Thus, generated samples with fixed cells were washed afterward, dried at the room temperature and stored in the refrigerator. The topography of fixed cells was obtained using the AFM system Ntegra Vita (NT-MDT, Zelenograd, Russia) working in the semi-contact mode with the silicon cantilever (Bruker, Mannheim, Germany) exhibiting spring constant of 0.1 N m^− 1^. The acquired images were processed and evaluated using the Nova and Gwyddion software packages, respectively.

### Statistical analysis

Data is presented as mean ± SD. One-way analysis of variance followed by Dunnet’s test was used for statistical evaluation. Differences were considered statistically significant at *p* < 0.05.

## Results and discussion

### Piezoelectric monitoring of exocytosis

**Fig. 2:**
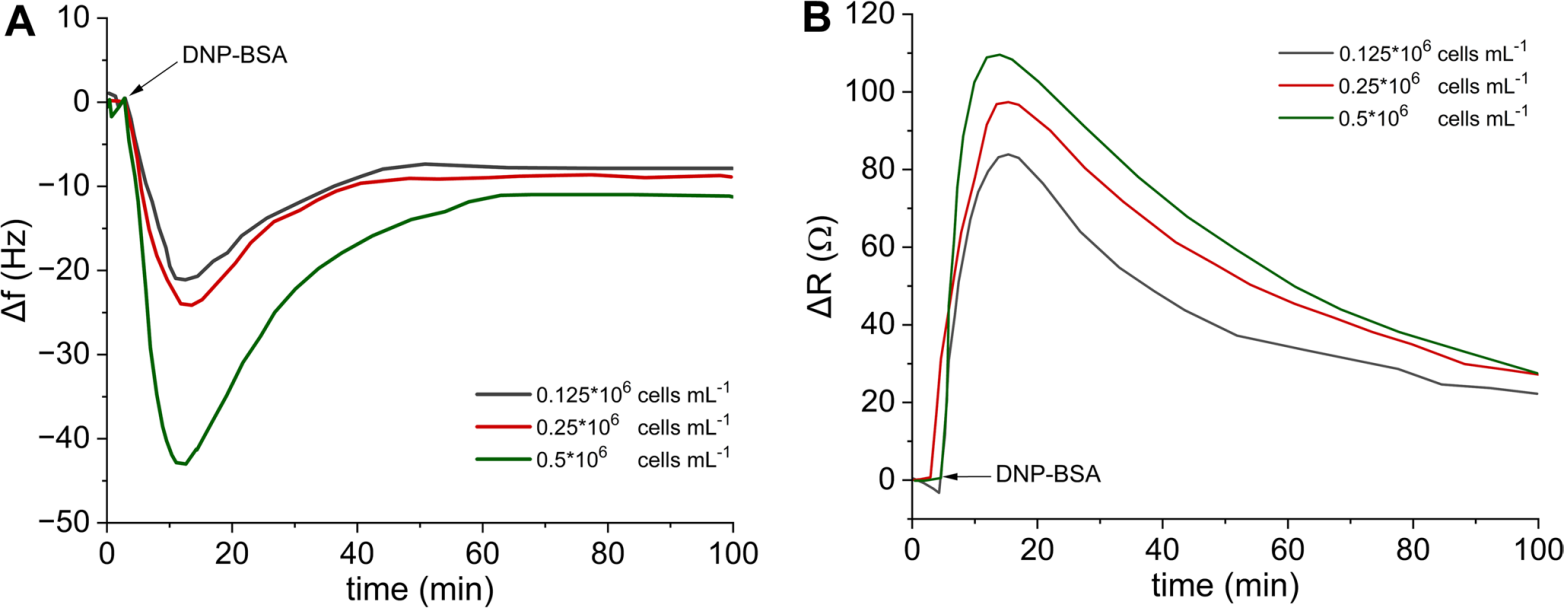
The frequency (**A**) and the resistance (**B**) raw responses of the piezoelectric sensor upon the DNP-BSA activation of various numbers of IgE-sensitized cells. The signal vs. time traces recorded after addition of DNP-BSA to the stabilized crystal with cells

The degranulation of mast cells is the process when the cells upon the antigen-induced crosslinking of IgE-FcεRI complexes release the granules from the cytoplasm to the extracellular space. This process as well as the final endocytosis of already formed complexes is accompanied by the change in the cell membrane and the cytoskeletal rearrangement [10, 11]. In this way, the cells realize both the recognition of the target analyte and generation of signal through microstructural changes on the sensing surface. Besides, this process appears to be dynamic and lasts quite a few minutes. Thus, antigen-induced exocytosis, monitored by PZ sensors, initially resulted in a decrease of frequency and an increase of resistance, taking basically some 10 min (Fig. 2A, B). Both shifts were attributed to the antigen induced exocytosis, associated with changes in membrane morphology and mechanical properties, including viscoelasticity, due to extensive cytoskeletal reorganization, granule fusion, and lipid remodeling. Several studies confirm increased cell adhesion and spreading, and membrane ruffling during exocytosis [33]. Vesicles fuse with the membrane, increasing surface area, which can transiently soften the membrane at early activation. Subsequently, the gradual increase of frequency and the slow decrease of resistance (> 30 min) attributed to the antigen-IgE-receptor like-endocytosis followed by the complex digestion and the cytoskeletal rearrangement to the original state [34]. 

To optimize the number of cells seeded on the PLL coated quartz crystal, experiments with various cell numbers were performed (Fig. 2A, B). Three concentrations of the IgE sensitized cells (0.5, 0.25 and 0.125 × 10^6^ cells mL^− 1^) were compared in ∆*f* frequency and ∆*R* resistance responses following the addition of DNP-BSA (0.1 µg mL^− 1^). By evaluating the ∆*f* and ∆*R* shifts at their maximum, it was expectedly concluded that both parameters increased with increased number of the initially seeded cells. The higher initial cell loadings did not improve the responses and technical problems with partial clogging of the flow-through system appeared. Apart from that, the concentration equal to 0.5 × 10^6^ cell mL^− 1^ resulted in the most sensitive responses and, thus, it was chosen for subsequent experiments.

**Fig. 3:**
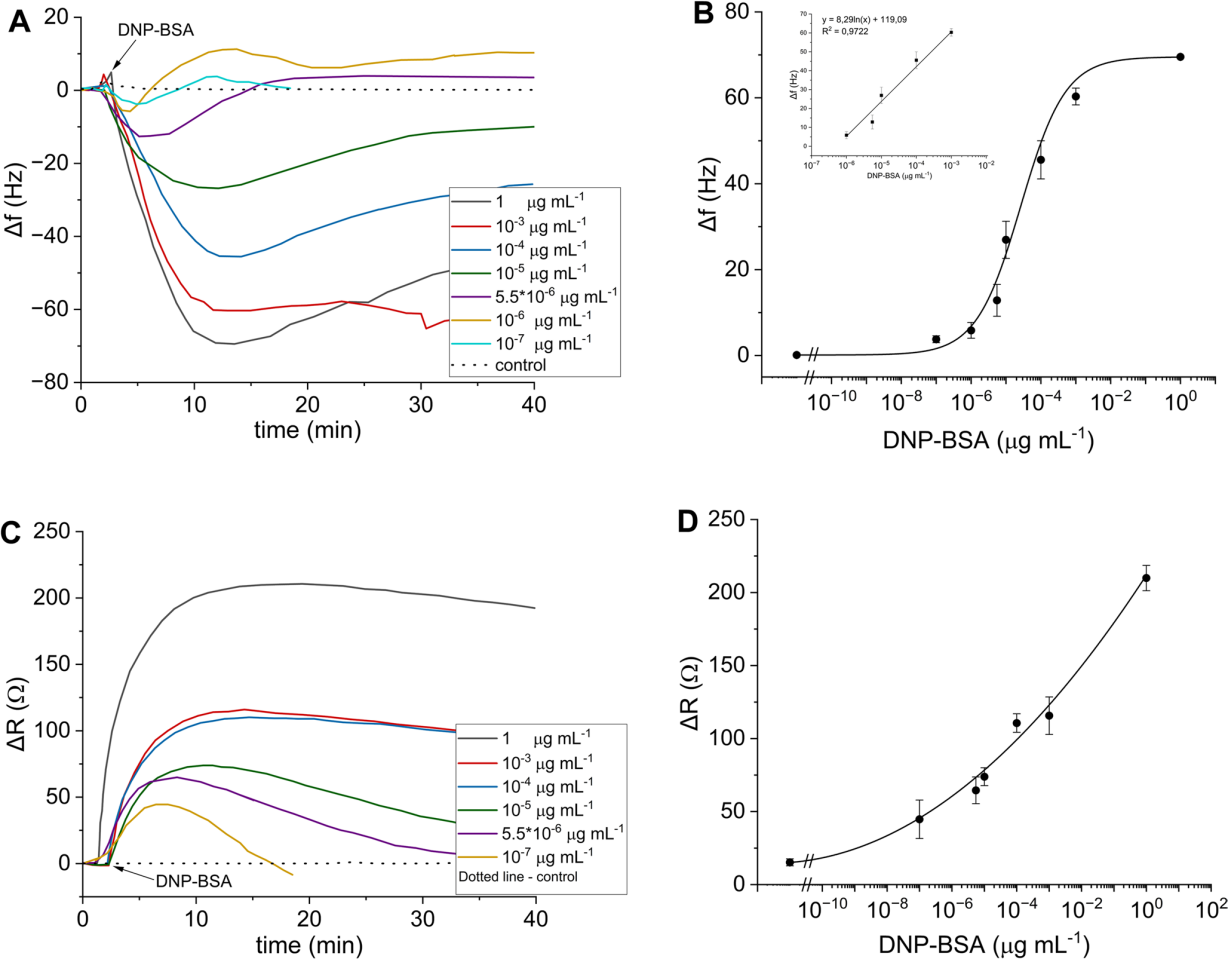
The frequency and resistance responses to various concentrations of DNP-BSA (**A**, **C**) are measured using the PZ sensors. The evaluated biosensor signals from time traces of frequency (**A**) and resistance (**B**) were converted to the plots of ∆*R* and ∆*f* versus concentration of antigen (**B**, **D**, respectively) from which (**B**) the IC_50_ value was estimated

Further, the concentration of antigen inducing the degranulation was optimized. The IgE-sensitized cells (0.5 × 10^6^ cell mL^− 1^) were seeded on the crystal overnight. The crystal holder was connected to the detector, and the frequency and resistance baselines were established under the constant flow of medium. Both frequency and resistance were recorded up to stabilization for various concentrations (10^− 7^ – 1 µg mL^− 1^) of DNP-BSA (Figs. 3A and C). The control experiment, involving the injection of the solution without antigen, did not influence any of the monitored parameters. The maximum in changes was plotted versus the corresponding concentrations of antigen (Figs. 3B and D). From the sigmoidal curve obtained by logistic fitting of data in Fig. 3B it is apparent that towards the higher concentrations of antigen, the frequency did not change significantly due to the antibody saturation by antigen. This result can be compared with the standard fluorescence assay for β-hexosaminidase published by Naal et al. – the Fig. 3A and B in [18] provide the β-hexosaminidase responses for similar DNP-BSA concentrations as utilized here. The estimated concentration of antigen 0.1 µg mL^− 1^ represents the maximal response after which the signal did not significantly change (PZ sensor) or decreased fluorescence [18] and importantly, it did not differ significantly within both methods. The EC_50_ value of ≈ 3.8 × 10^− 5^ ± 1.1 × 10^− 5^ µg mL^− 1^ of DNP-BSA and working range from (3.16 ± 0.94) x 10^− 6^ to (4.6 ± 2.0) x 10^− 4^ µg mL^− 1^ were obtained from the logistic function of frequency plotted experiment as interval between *EC*_20_ and *EC*_80_. Limit of detection (LOD) was estimated to 3.3 × 10^− 7^ µg mL^− 1^ (based on the S/*N* = 3 ratio). Looking at the same graphs with resistance signal (Fig. 3D), the observed trend was probably due to the still existing viscoelastic changes of cells. In this work, the resistance *R* is utilized alternatively to the dissipation parameter *D* provided in alternative commercial instruments [35]. The observed change of resistance results from complex morphological changes associated with the degranulation process; the viscoelastic changes can be accompanied by changes of the surface density due to the formation of vesicles and granules, as characterized below using the AFM imaging.

**Fig. 4:**
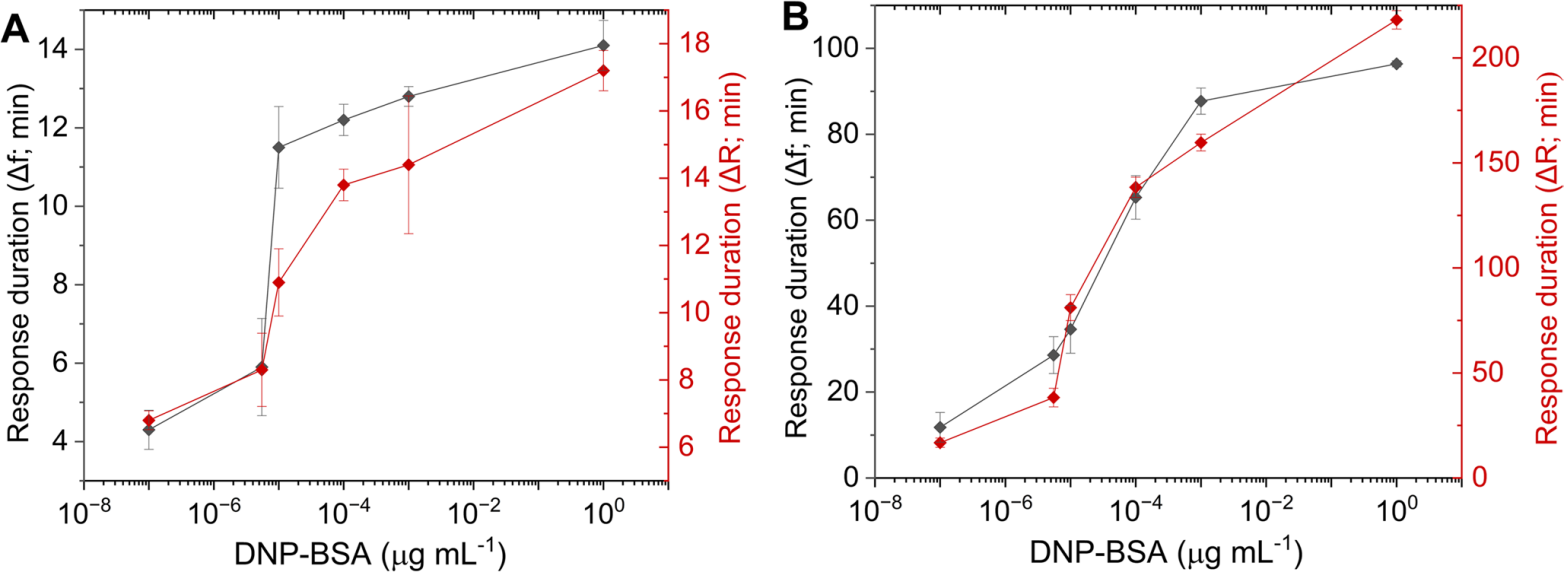
The kinetics of PZ responses of IgE/RBL- 2H3 cells stimulated by DNP-BSA. The frequency and resistance time response to peak amplitude (**A**) and the estimation of response duration after stimulation (**B**). The times for the peaks (**A**) and duration of the effect (**B**) were obtained from the curves presented in Fig. 3A and C, respectively

In Figs. 4A, B, the kinetics of the PZ responses are shown for the IgE-sensitized RBL-2H3 cells stimulated with the antigen. The increasing times for both peak amplitude and duration of response (based on the returns of the signals Δ*f* and Δ*R* to initial levels as shown in Fig. 3A and C, respectively) corresponded with the extension of the cytoskeletal rearrangement and spreading towards the increasing concentrations of the bound activating antigen. The detected levels of antigen were extremely low; based on the monitoring of resonance frequency in Fig. 3A, the effect of 10^− 7^ µg mL^− 1^ was clearly visible and even lower levels of antigen caused measurable changes of resonance of the piezosensor.

Similar data were published by Abbasi et al. [28]. when the stationary impedance-based measurement of degranulation was used. Based on the rhodamine-phalloidin visualization of actin filaments, the extensive membrane ruffling was apparent as early as 2.5 min after the addition of antigen, followed by spreading of cells and formation of lamellipodia. The cytoskeletal reorganization observed by Abbasi reached the maximum at 30–45 min after the addition of antigen and it returned to the spindle shape morphology 4 h later which correlated with the impedance response. Here, frequency and resistance changed independently but for the 0.1 µg mL^− 1^ concentration of antigen, the time to the peak amplitude for resistance was estimated at 15 min and the duration of the response was around 3 h.

### Measurement of inhibition of degranulation

**Fig. 5:**
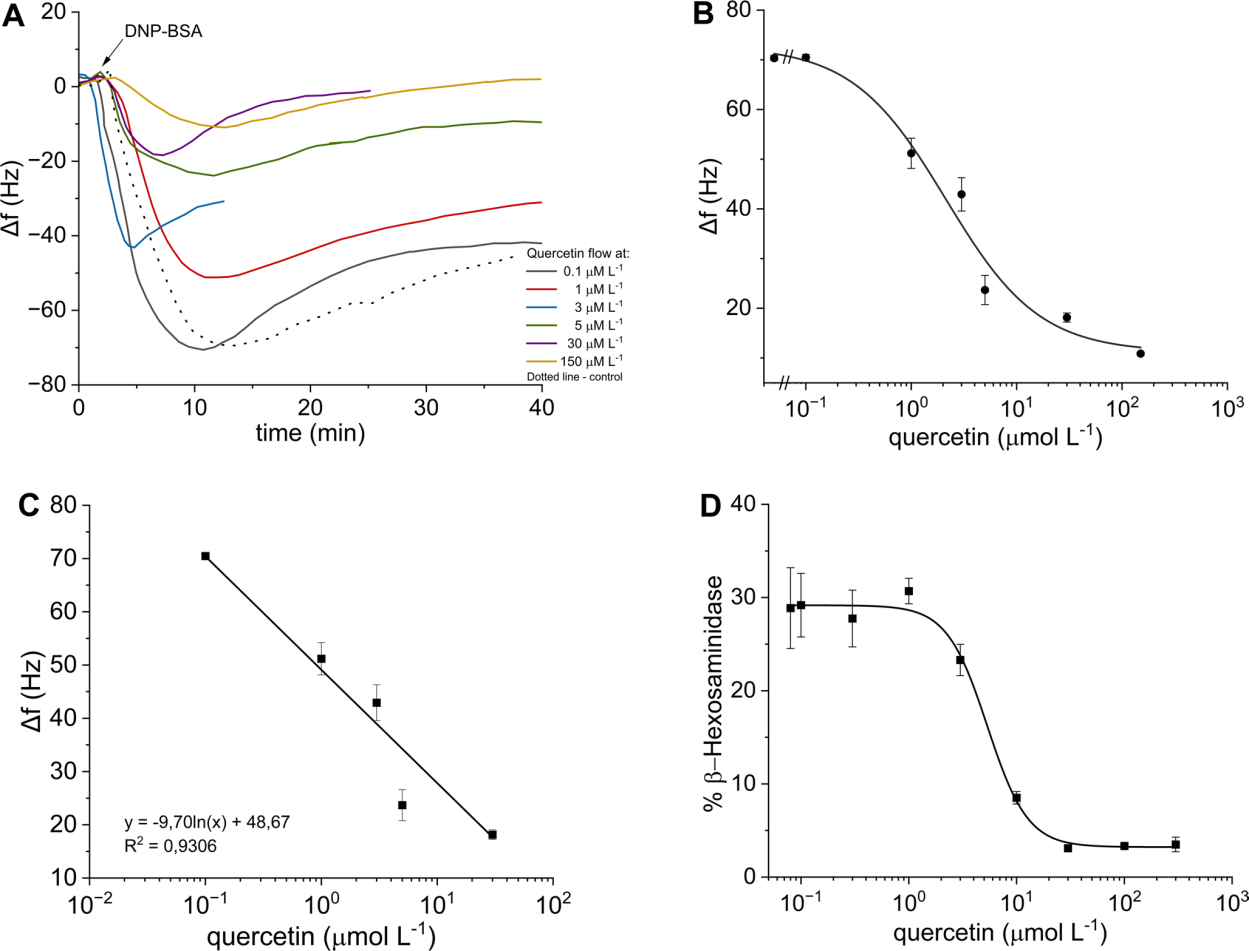
The concentration dependent inhibition of the degranulation of RBL – 2H3 cells by quercetin described by the change of frequency (**A**). PZ response vs. concentration of quercetin (**B**, **C**) and standard fluorescence assay (**D**)

Quercetin is a well-known inhibitor of mast cell degranulation, and as with the majority of effective or potentially tested anti-allergic substances, its solubility in water is poor. Quercetin was diluted in the MEM medium to final concentrations from 0.1 to 150 µmol L^− 1^. The content of DMSO was always kept below 0.01%, which does not influence the cellular activity as confirmed previously in the fluorescence assay [18] and also reported elsewhere [36]. Thus, the continual flow of medium supplemented with quercetin through the chamber was established. As soon as the monitored signal appeared stable, the DNP-BSA antigen at the fixed 0.1 µg mL^− 1^ concentration was injected into the carrier medium flowing through the chamber and the ∆*f* was monitored (Fig. 5A).

The estimated IC_50_ value of quercetin from logistic fit was 2.7 ± 1.1 µmol L^− 1^; it determines the concentration required to inhibit half of the maximum biological response shown in Fig. 5B, C. The working range between 0.48 ± 0.36 µmol L^− 1^ and 15.07 ± 7.55 µmol L^− 1^ was evaluated from logistic function as interval between EC_20_ and EC_80_. LOD was estimated near 0.1 µmol L^− 1^. Thus, data shows that the lowest concentration of quercetin did not inhibit at all as the response to the addition of antigen corresponded with the frequency response of 0.1 µg mL^− 1^ DNP-BSA (dotted line in Fig. 5B).

Using the common inhibition experiment carried out by the standard fluorescence assay, the IC_50_ value of 5.35 µmol L^− 1^ and working range of 2.90–9.70 µmol L^− 1^ were obtained from the logistic fit (Fig. 5D). Even if both IC_50_ differ slightly, it is unwise to make an exact comparison between the QCM technique and standard fluorescence assay due to the distinct character of the measurement as well as cellular parameters being detected. Overall, the final observed effect of quercetin – inhibition of degranulation of mast cells, was consistent with previous studies [22, 23, 25]. However, the proposed PZ biosensor has the potential of increased sensitivity compared to the conventional fluorescence methods for drug screening.

### Characterization of exocytosis using AFM

**Fig. 6:**
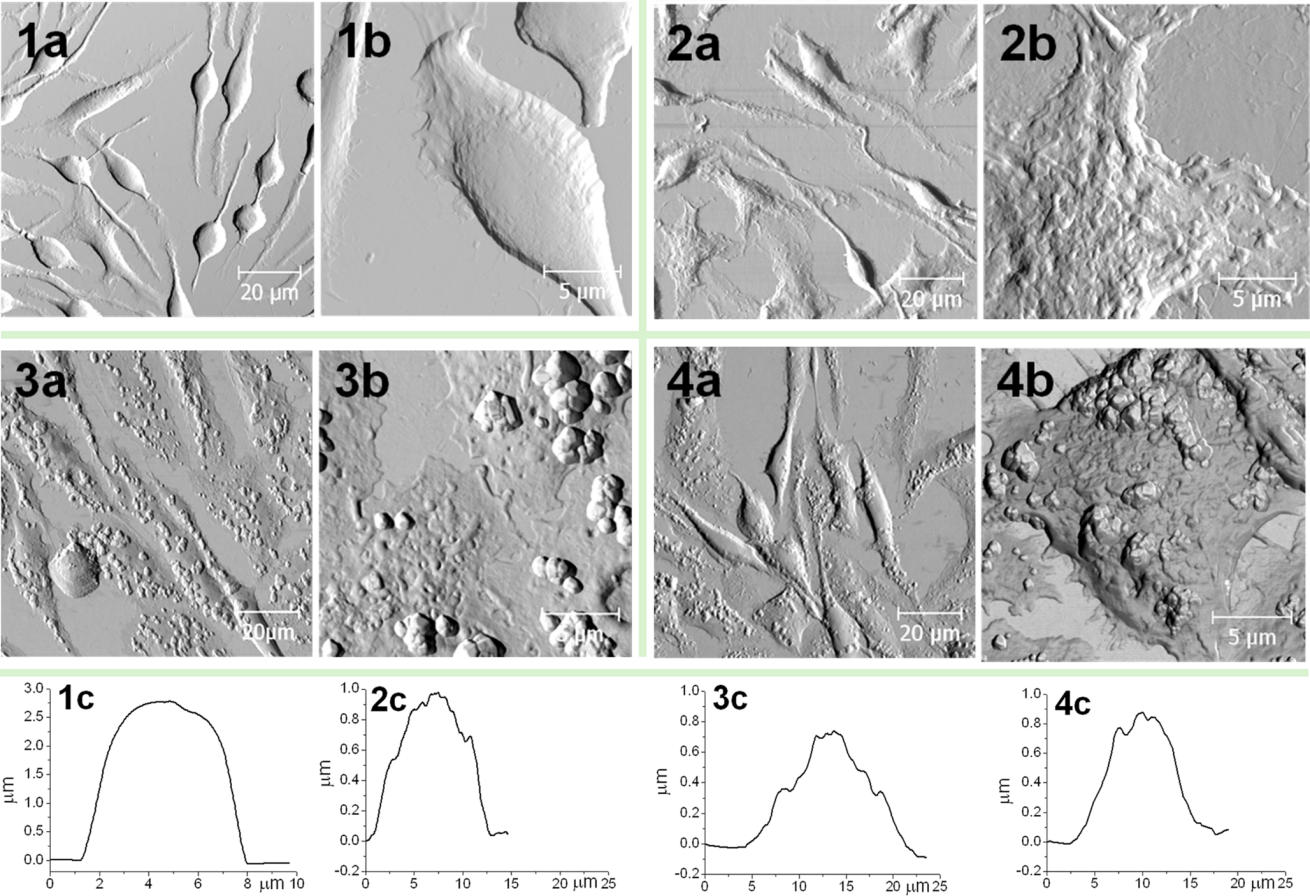
The topography of fixed RBL – 2H3 mast cells upon the antigen – induced degranulation shown as large scale (**a**) and zoomed (**b**) AFM images. The control native cells before the addition of antigen (1) and the antigen activated cells fixed at 30 s (2), 10 min (3) and 40 min (4) after the initiation of activation. The vertical height profiles of the cell (**c**) were generated from the zoomed images (**b**)

The fixed RBL-2H3 cells were examined to visualize both the membrane changes and granule-like structures upon the antigen activation using AFM. The height and width of the fixed cells were estimated since the degranulation is expected to make the cells more adherent and spread on the surface. Figure 6 shows the control experiment and the time-dependent degranulation of fixed RBL-2H3 cells. Looking at the control (Fig. 6, row 1, images *a* and *b*), the round-shaped cells and some well-spread cells occurred, and the cell membrane appeared almost smooth. Considering the activated cells at 30 s after the antigen activation (Fig. 6, row 2 *a*,* b*), the cells became more spread with the characteristic ruffling of the cell membrane that may hide the exocytotic pores. The prolongation of the activation led to the clear visualization of granules released from the cells (Fig. 6, row 3 *a*,* b*). At the time of 40 min, the degranulated cells were still found, even if it became obvious that the cells were slowly returning to their original state (Fig. 6, row 4 *a*,* b*). By estimating the height and the width of cellular body, it was possible to compare both parameters for stimulated and non-stimulated cells. The control cells were *h* = 3 ± 0.5 μm high and *w* = 7 ± 1 μm wide (Fig. 6, image 1*c*). Contrary, the gradual exocytosis changed these parameters to *h* = 1 ± 0.1 μm and *w* = 12 ± 0.5 μm (30 s; Figs. 2c and 6); *h* = 0.8 ± 0.1 μm and *w* = 17 ± 1.5 μm (10 min; Figs. 3c and 6); *h* = 0.9 ± 0.1 μm and *w* = 15 ± 1 μm (40 min; Figs. 4c and 6). Thus, there is an evident change in morphology of exocytotic cells upon the activation.

Similar granule-like structures were observed in previous report on the peritoneal mast cells stimulated with compound 48/80 or bone marrow-derived mast cells stimulated with DNP-BSA [37, 38]. However, the common features of activated cells such as spreading and lamellipodia formation occur but it is obvious that the type of mast cells and the compounds/antigen causing the degranulation can play significant role, too.

The control cells (Fig. 6, row 1 *a*,* b*) did not spread on the surface as extensively as the activated ones and the cell membrane of the controls appeared smoother. On the other hand, the time-dependent images of the activated cells were associated with membrane ruffling (Fig. 6, row 2 *a*,* b*), cell spreading and granule-like structures attached to the cell membrane (Fig. 6, rows 3–4 *a*,* b*).

## Conclusions

The biosensor based on piezoelectric detection was developed for the real-time monitoring of exocytosis and degranulation of the RBL-2H3 mast cells responding to the added antigen. The mast cell line functions both as the specific biorecognition element and as system generating signal originating from the morphological degranulation changes triggered by the presence of target analyte. Thus, the native behavior of this cell line was successfully utilized for complex biosensing. The resulting biosensor was optimized regarding the number of cells and concentration of the activating antigen; the LOD value of 3.3 × 10^− 7^ µg mL^− 1^ of DNP-BSA and working range from ≈ 4.6 × 10^− 4^ to 3.2 × 10^− 6^ µg mL^− 1^ were obtained from the PZ frequency plot. Furthermore, the effect of quercetin-induced inhibition of degranulation was sensitively monitored, providing sigmoidal response curve characterized by a low IC_50_ value (≈ 2.7 µmol L^− 1^) compared to the standard hexaminidase activity fluorescent assay (5.4 µmol L^− 1^). Limit of detection of the inhibitor was estimated to 0.1 µmol L^− 1^. The expected changes of cell morphology were confirmed by independent atomic force microscopy scans of the cells fixed at defined time intervals after the activation event. In future, the proposed piezoelectric cell-based biosensor will be tested for screening newly discovered compounds with potential antiallergic activity. The coupling of mast cells on the piezoelectric transducers can provide the convenient “cells on chip” concept in the future where the biological events can be monitored fast, sensitively and in real time. Moreover, the PZ technique is a label-free approach compared to fluorescence, which can affect the normal physiological state of the cells.

## Data Availability

Available from the corresponding authors upon request.
